# A Bovine Cell Line That Can Be Infected by Natural Sheep Scrapie Prions

**DOI:** 10.1371/journal.pone.0117154

**Published:** 2015-01-07

**Authors:** Anja M. Oelschlegel, Markus Geissen, Matthias Lenk, Roland Riebe, Marlies Angermann, Hermann Schaetzl, Martin H. Groschup

**Affiliations:** 1 Institute of Novel and Emerging Infectious Diseases at the Friedrich-Loeffler-Institut, Greifswald—Isle of Riems, Germany; 2 Project Group Neuropharmacology, Leibniz Institute for Neurobiology, Magdeburg, Germany; 3 Department of Vascular Medicine, University Heart Centre Hamburg, UKE, Hamburg, Germany; 4 Department of Experimental Animal Facilities and Biorisk Management at the Friedrich-Loeffler-Institut, Greifswald—Isle of Riems, Germany; 5 Administrative District Office Goerlitz, Goerlitz, Germany; 6 Department of Comparative Biology and Experimental Medicine, Faculty of Veterinary Medicine, University of Calgary, Calgary, Canada; Rocky Mountain Laboratories, NIAID, NIH, UNITED STATES

## Abstract

Cell culture systems represent a crucial part in basic prion research; yet, cell lines that are susceptible to prions, especially to field isolated prions that were not adapted to rodents, are very rare. The purpose of this study was to identify and characterize a cell line that was susceptible to ruminant-derived prions and to establish a stable prion infection within it. Based on species and tissue of origin as well as PrP expression rate, we pre-selected a total of 33 cell lines that were then challenged with natural and with mouse propagated BSE or scrapie inocula. Here, we report the successful infection of a non-transgenic bovine cell line, a sub-line of the bovine kidney cell line MDBK, with natural sheep scrapie prions. This cell line retained the scrapie infection for more than 200 passages. Selective cloning resulted in cell populations with increased accumulation of PrP^res^, although this treatment was not mandatory for retaining the infection. The infection remained stable, even under suboptimal culture conditions. The resulting infectivity of the cells was confirmed by mouse bioassay (Tgbov mice, Tgshp mice). We believe that PES cells used together with other prion permissive cell lines will prove a valuable tool for ongoing efforts to understand and defeat prions and prion diseases.

## INTRODUCTION

Scrapie is considered to be the archetype of transmissible spongiform encephalopathies (TSE) or prion diseases, a group of fatal neurodegenerative disorders that received considerable public and scientific attention due to a widespread bovine spongiform encephalopathy (BSE) epidemic in cattle in the United Kingdom and elsewhere, and after it was shown that BSE causes a variant form of Creutzfeldt-Jakob disease in humans. Other closely related neurodegenerative protein misfolding diseases include Alzheimer’s disease, Parkinson’s disease and Huntington’s disease.

According to the prion hypothesis [1] an abnormally folded isoform (PrP^Sc^ or PrP^res^) of the endogenous, cellular prion protein (PrP^C^) is the sole component of the infectious agent, the prion. Recombinant prions have been generated [2]. The protein misfolding cyclic amplification (PMCA) [2–4] has been developed to simulate the refolding and growth of aggregated prion protein in-vitro. Numerous conventional and transgenic rodent models have been established to isolate, quantify and characterize cattle- or small-ruminant-derived BSE and scrapie prions [5, 6]. A major disadvantage of these *in-vivo* systems is that BSE and scrapie prions, even when adapted to rodents, induce long incubation times of several months or years. Furthermore, these experiments require the sacrifice of numerous animals, and they are cost intensive. For many years it has therefore been a prime objective in prion research to establish prion susceptible cell lines. Cell culture models combine the rapidness of a system that is characterized by short generation cycles with the complexity of an *in vivo* model. Prion infected cell lines can be used to study the cell biology of the physiologically and the abnormally folded prion protein, as well as the characteristics of different prion strains [7]. Cell culture models can facilitate basic as well as diagnostic prion research and last but not least they can be used to screen for potential therapeutic drugs; for ref. see [8].

The very first attempts to obtain TSE infected cell lines were made already in 1965 [9]. In 1970 the first prion propagating cell line was derived from a scrapie (mouse adapted scrapie) infected mouse [10, 11] and in 1976 Clarke and Milson succeeded to actually infect murine fibroblastoma cells with mouse scrapie prions [12]. In the following years further neural and non-neural murine cell lines were reported to be prion susceptible, however their susceptibility was restricted to a rather small number of different mouse adapted prion strains. To date the murine neuroblastoma cell line N_2_a and several N_2_a-derived sub-lines are the most frequently used cell culture systems for experimental prion strain propagation [7, 13–23]. In 1984 it was demonstrated that rat cells could be infected with mouse adapted 139A scrapie prions [24, 25], and in 1990 hamster cells were infected with experimental 263K hamster scrapie prions [26]. Later it was shown that also neural embryonic stem cells might provide a model for murine prion strains [27, 28]. Transgenic Rov cells [29], rabbit epithelial cells (RK13) that overexpress ovine PrP^C^, were the first cells found to be susceptible for natural sheep scrapie prions. This finding was followed by several reports about other transgenic cell lines—either also based on RK13 cells or on others—propagating prions of various strains matching the PrP^C^ that they expressed [18, 19, 30, 31]. Finally in 2006, Raymond et al. published a transformed deer cell line that had been successfully infected with Chronic Wasting Disease [32].

Much is still left to learn about the infectious nature of prions and the factors that determine the intrinsic susceptibility of the host. It is astonishing that most cell lines seem to be resistant to prion infection [15, 24], whereas mouse, sheep and cattle are susceptible to the disease, develop clinical signs and die. With regard to natural sheep scrapie or BSE prions the infection of cell lines seems to be particularly difficult. Only a few cell lines are prion susceptible and this susceptibility is limited to very specific prion strains. Only a certain percentage of cells within a culture gets actually infected [14, 33, 34]. Frequently this infection is not stable, but is lost after a certain period of time [34–36]. Until today, it was not possible to infect and establish a non-transgenic cell line with natural sheep scrapie or natural BSE prions. Is this due to a specific feature of the natural TSE isolates or to the cell lines? Is it a matter of host-agent-correspondence? Are there other factors that support or handicap the prion propagation?

The main focus of this study was the identification of a non-transgenic cell line that is susceptible to bovine or ovine prions. To this purpose we pre-selected 54 promising candidate cell lines from a cell line collection of about 1400 cell lines, which is hosted at the Friedrich-Loeffler-Institut (FLI). The cell lines originated mainly from sheep, goats or cattle, represented various cell types, and 33 of them expressed cellular prion protein at high levels. We challenged these 33 cell lines with different natural BSE and scrapie field isolates as well as with experimental mouse propagated BSE and scrapie strains. By doing so, we identified, characterized and established a bovine cell line that is susceptible to natural sheep scrapie prions.

## MATERIALS AND METHODS

### Cells and Cell culture

All test cell lines were obtained from the collection of cell lines in veterinary medicine (CCLV) of the FLI (Table 1). The cells were cultured at 37°C and 5% CO_2_ in Opti-MEM (Gibco) supplemented with 10% fetal calf serum (FCS) (Invitrogen) and 1% P/S (penicillin: 126 µg/ml [Sigma-Aldrich], streptomycin: 125 µg/ml [Sigma-Aldrich]). Upon reaching confluence cells were split at cell line specific ratios. RML infected ScN_2_a cells [14, 15], kindly provided to us by S. Priola (Hamilton, MT), were cultured at 37°C and 5% CO_2_ in Opti-MEM supplemented with 10% FCS and 1% P/S and were maintained by 1:2 splits. RML infected SMBRC040 cells (TSE Resource Centre) [10, 11] were cultured at 34°C in MEM199 (Gibco) supplemented with 5% FCS, 10% NCS (Invitrogen) and 1% P/S and were maintained by 1:3 splits.

**Table 1:** Selected cell repository test cell lines.

**Name (CCLV RIE reference number)**	**Species**	**Age**	**Tissue**	**Split ratio**	**PrP^C^**	**Inocula^#^**
KM-2-R (89)	cattle	fetus	spleen	1:2	+	BSE
KZ-R (93)	cattle	fetus	tongue	1:2	+	BSE
KNN-R (96)	cattle	fetus	adrenal gland	1:2	+	BSE
KMU-R (98)	cattle	neonate	muscle	1:2	-	
PES (154)	cattle	adult	kidney, BVDV+	1:2	+	BSE, **scrapie**, RML
KHY (239)	cattle	neonate	pituitary gland	1:2	+	BSE, RML, ME7
KOP-R (244)	cattle	neonate	oesopharynx	1:2	+	BSE
MDBK (84)	cattle	adult	kidney, BVDV+	1:6	+	BSE, scrapie
MDBK (261)	cattle	adult	kidney	1:6	+	BSE, scrapie
GGE-R (456)	cattle	embryo	brain	1:2	+	BSE, RML, ME7
GRE-R (458)	cattle	embryo	brain	1:2	+	*BSE*, scrapie, RML, ME7
FKD-1-R (970)	cattle	fetus	gut	1:2	+	BSE
FP-R (972)	cattle	fetus	rumen	1:2	+	BSE
FRD-R (979)	cattle	fetus	gut	1:2	+	BSE
PT (11)	sheep	adult	kidney	1:6	-	BSE, scrapie
PO (17)	sheep	adult	kidney	1:6	-	
SFN-R (39)	sheep	neonate	kidney	1:2	+	scrapie
SFH-R (42)	sheep	neonate	testis	1:2	-	
SFT-R (43)	sheep	neonate	thymus	1:3	-	
SPLW-R (150)	sheep	lamb	plexus chorioideus	1:6	-	
SLU-L (151)	sheep	adult	pulmonary tumor	1:6	+	scrapie
OFC (157)	sheep	fetus	cornea	1:2	-	
SE-R (174)	sheep	embryo	embryo	1:2	-	
ESP (201)	sheep	embryo	plexus chorioideus	1:2	+	BSE, scrapie, RML, ME7
SCP (213)	sheep	embryo	plexus chorioideus	1:2	+	BSE, scrapie, RML, ME7
SP-R (279)	sheep	adult	plexus chorioideus	1:2	-	
ZLU-R (120)	goat	fetus	lung	1:2	+	BSE, scrapie, caprine scrapie
ZLI-R (130)	goat	fetus	labium	1:2	+	BSE, scrapie, caprine scrapie
ENG-R (242)	mink	embryo	brain	1:2	+	BSE, ME7, RML
KG-R (252)	cat	neonate	brain	1:2	+	BSE, ME7, RML
WSG-R (378)	wild pig	fetus	brain	1:4	+	BSE
CBS (266)	domestic pig	fetus	cerebellum	1:4	+	BSE
SGW-R (565)	domestic pig	weaned	brain stem	1:4	+	BSE, ME7, RML
DWM-R (766)	fallow deer	embryo	stomach	1:2	-	BSE
REH-R (822)	roe deer	fetus	testis	1:2	-	BSE
REN-R (821)	roe deer	fetus	kidney	1:2	-	
RPS-R (824)	roe	fetus	mucosa of rumen	1:2	-	
HEK-293 (197)	human	embryo	kidney	1:4	+	BSE
Oligo (221)	human	embryo	oligodendroglia	1:4	+	
K562 (439)	human	unknown	myeloid leukemia	1:4	+	
U373 (675)	human	adult	astrocytoma	1:2	+	BSE
L,NCTC/L-929 (14)	mouse	adult	connective tissue	1:6	+	BSE, RML
N2a (132)	mouse	adult	neuroblastoma	1:3,1:10	+	RML
N2a (229)	mouse	adult	neuroblastoma	1:3	+	BSE, scrapie, **RML**
M0/0 (864)	mousePrP^0/0^	neonate	brain	1:2,1:10	-	BSE
MGbov (900)	mousePrP^0/0^PrP^bov^	embryo	brain	1:3	+	*BSE*, scrapie, RML, murine BSE
MGOV-R 23a (969)	mousePrP^0/0^PrP^ov^	embryo	brain	1:4	+	BSE, scrapie, RML
MGOV-R 28 (969)	mousePrP^0/0^PrP^ov^	embryo	brain	1:3	+	scrapie, RML
CHO-K1 (134)	hamster	adult	ovary	1:6	+	BSE, RML
RK-13 (109)	rabbit	adult	epithelial cells	1:4	-	
RLI-R (750)	rabbit	adult	liver, immortalized	1:6	-	
JKM (795)	rabbit	young	spleen	1:2	-	
T34 (830)	rabbit	adult	spleen	1:2	-	
KMK-R (901)	rabbit	adult	bone marrow	1:2	-	

^#^ **bold**: successful infection; *italic*: transient infection, but not repeatable (data not shown)

### Inocula and Inocula preparation

Inocula were prepared freshly on the day of the first inoculation. Inocula were prepared from: mouse adapted scrapie strains (RML, ME7), a mouse adapted BSE isolate (BSE-UK-A-26 in Tgbov XV mice), the pooled brains of five cattle infected with classical BSE (R13/04, R14/04, R18/04, R6/07, R12/07), two atypical BSE isolates (H-type: R152/04 and L-type: R172/02), classical and atypical scrapie isolates derived from sheep that carried different genotypes (classical: S31/03[ARQ/ARQ], S33/03[ARQ/ARQ], S11/04[ARQ/ARQ], S66/04[ARQ/ARQ], 67/04[ARQ/ARQ], 68/04[ARQ/ARQ], 69/04[ARQ/ARQ], 70/04[ARQ/ARQ], 71/04[ARQ/ARQ], S72/04[ARQ/ARQ], S73/04[ARQ/ARQ], S74/04[ARQ/ARQ], S75/04[ARQ/ARQ], S76/04[ARQ/ARQ], S77/04[ARQ/ARQ], S79/04[ARQ/ARQ], S80/04[ARQ/ARQ], S91/04[ARQ/ARQ], S92/04[ARQ/ARQ], S93/04[ARQ/ARQ], S94/04[ARQ/ARQ], S95/04[ARQ/ARQ], S96/04[ARQ/ARQ], S101/04[ARQ/ARQ], S102/04[ARQ/ARQ], S107/04[ARQ/ARQ], S120/04[ARQ/ARQ], S126/04 [ARQ/ARQ], S1/06[ARQ/ARQ], S3/07[ARQ/ARQ], S24/07[ARQ/ARQ], S25/07[ARQ/ARQ], S26/07[ARQ/ARQ], S27/07[ARQ/ARQ], S90/04[AHQ/ARQ], S100/04[AHQ/ARQ], S13/04[VRQ/ARH], S6/06[ARQ/VRQ], S12/04[ARQ/VRQ], S52/04[ARQ/VRQ]; atypical: S27/05[ARR/AHQ], S16/06[ARR/ARR]) or a caprine scrapie isolate. Brain homogenates (10% [wt/vol]) were prepared in sterile phosphate buffered saline (PBS; 140 mM NaCl, 2.7 mM KCl, 6.5 mM Na_2_HPO_4_ x 2H_2_O, 1.5 mM KH_2_PO_4_, pH 6,9) or sucrose-buffer (5% [wt/vol] sucrose in ddH_2_O) and then sonicated for two minutes. An aliquot was stored at-20ºC for a second cell application three days later.

### Inoculation of cells

One day prior to inoculation, cells were seeded to reach approximately 80% confluence the next day. On the following day, the cell supernatant was removed and replaced with fresh culture medium containing the prepared brain homogenate (final concentration 0.33% [wt/vol]). After an incubation period of three days, the supernatant was replaced by new inoculum (0.33% [wt/vol]) in fresh culture medium and another three days later fresh medium was added to the existing cell supernatant (the extended exposure time was a concession to the possibility that ruminant-derived BSE and scrapie field isolates might not be as infectious as rodent passaged scrapie prions). After an overall incubation period of nine days, the cell supernatant was removed and, following extensive washing with PBS, replaced with fresh culture medium. Cells were further cultivated and passaged repeatedly at cell line specific split ratios (Table 1), thereby eliminating residual inoculum up to a cumulative dilution of 1:1000 [22]. During each split, cell aliquots were stored away. Later these samples were used to verify the removal of the inoculum and to possibly detect cell propagated PrP^res^.

### Detection of PrP by western-blot analysis

Cell pellets were obtained by centrifugation at 1250 x g for 10 min, and 10% (wt/vol) lysates were prepared by resuspending the cell pellets in lysis buffer (50 mM Tris-HCl, pH 8.0/ 150 mM NaCl/ 0.5% sodium deoxycholate/ 0.5% TritonX-100). The lysates were centrifuged a second time (1250 x g; 10 minutes). The supernatants were incubated with 3.3 µg RNaseA (Sigma-Aldrich)/ml and 100µg DNaseI (Roche)/ml at 37°C for one hour. For the specific detection of PrP^res^ this was followed by one hour of incubation with 25 µg ProteinaseK (PK) (Sigma-Aldrich)/ml at 37°C; non PK-treated controls were assayed in parallel. The PK digestion was stopped with 10 µl PMSF (stock 100 mM)/ml. Loading buffer (10x CVL: 10% SDS/ 0.25 M Tris-HCl/ 25% ß-mercaptoethanol/ 15% sucrose/ 0.05% bromphenolblue/ pH 6.8) was added and the samples were sonicated (20 seconds) and denatured (90°C, 5 minutes). Optionally the protein content of the samples was concentrated by ultracentrifugation (281595 x g/ 4°C/ 1 hour) and the samples were resuspended in 2x CVL loading buffer. Electrophoretic separation and semi-dry transfer (BioRad) to polyvinylpyrrolidon-membranes (Millipore) were performed using standard procedures. Blots were incubated in blocking solution (5% non-fat dry milk in PBST [PBS; 0.1% Tween20]) for one hour and immunostained with a monoclonal anti-PrP antibody (ICSM18 [D-Gen] 1:10000, 6H4 [Prionics AG] 1:10000, P4 hybridoma supernatant [37] 1:1000, L42 hybridoma supernatant [37] 1:100), followed by three washes with PBST and one hour incubation with an HRP-conjugated secondary antibody. Following three washes with PBST, chemiluminescence was induced by ECL (Amersham) and visualized by X-ray-film (Amersham) or VersaDoc^TM^ (BioRad).

### Detection of PrP by dot-blot analysis

The dot-blot technique was used in a modified way as described by Geissen et al. [38]. Cells were lysed as described above and transferred (100–150 µl/well) onto a polyvinylpyrrolidon-membrane (activated in methanol and equilibrated in lysis buffer) in a dot-blot-apparatus (Roth) by applying vacuum. The membrane was dried for one hour at 37°C, incubated with 100 µg DNaseI/ml for one hour, followed by incubation with 25 µg PK/ml at 37°C for 90 minutes. The membrane was then rinsed twice with ddH_2_O, incubated with 3M guanidinium thiocyanate (in 10 mM Tris-HCl; pH 8.0) for 10 minutes, rinsed five times with water and incubated for one hour with blocking solution (5% non-fat dry milk in PBST). Immunostaining and detection was done as described for western-blots.

### Detection of PrP by Cell-ELISA

The Cell-ELISA-technique was used in a modified version as described by Hoelscher et al. [39]. Cells were fixed in a cell line dependent manner for 30 minutes up to one hour with 5% (wt/vol) paraformaldehyde (in PBS, 1 mM MgCl_2_), rinsed with PBS (PBS, 1 mM MgCl_2_) and permeabilized with 0.4% (vol/vol) TritonX-100 (in PBS) for 3 minutes. For the selective detection of PrP^res^, cells were digested for 15 minutes with 25 µg PK/ml. The reaction was stopped by incubation with 2 mM PMSF (in PBS) for 15 minutes, followed by denaturation with 6 M guanidinium hydrochloride (in 50 mM Tris-HCl; pH7.4) and two washes with TNT (150 mM NaCl, 10 mM Tris, 0.05% Tween20). After incubating the cells in blocking solution (5% non-fat dry milk in TNT), immunostaining was performed over night with different monoclonal antibodies (ICSM18 [D-Gen] 1:5000, 6H4 [Prionics AG] 1:5000, P4 hybridoma supernatant [37] 1:100, L42 hybridoma supernatant [37] 1:50) followed by an Alkaline Phosphatase (AP) coupled secondary antibody. Positive signals of PrP^res^ were detected with the AP Conjugate Substrate Kit (Biorad). The evaluation was done using a standard cell culture invert microscope (Zeiss).

### Cell-subcloning

To obtain single cell clones, 200 cells were seeded into 10-cm-cell-culture-dishes. Cell colonies consisting of sister cells were isolated by gentle pipetting, transferred to 96-well plates and expanded further.

### PK resistance

**Time series**. Cell lysates were prepared as described for western-blot analysis. DNaseI/RNaseA digested samples were divided into 13 aliquots, supplemented with ProteinaseK (25 µg/ml) and incubated at 37°C. In intervals of two hours the digestion of one aliquot at a time was stopped by adding 10 µl PMSF (100mM)/ ml. Further sample preparation and detection of PrP^res^ was done according to dot-blot or western-blot techniques. **Concentration series**. Cell lysates were prepared as described for western-blot analysis. DNaseI/RNaseA digested samples were divided into six aliquots, supplemented with increasing concentrations of ProteinaseK (0 µg/ml, 25 µg/ml, 100 µg/ml, 250 µg/ml, 500 µg/ml and 1000 µg/ml) and incubated for one hour at 37°C. The digestion was stopped with 10 µl PMSF (100 mM stock)/ ml. Further sample preparation and detection of PrP^res^ was done according to dot-blot or western-blot techniques.

### PrP^res^ inhibiting substances

Imatinib [40] (Glivec; Novartis), Pentosansulfat [41] (Fibrezym; bene Arzneimittel GmbH) and Suramin [42] (Sigma-Aldrich) were used to decrease PrP^res^ in infected cells. The substances were diluted in culture medium and applied to the cells during culture (Imatinib 10 µM, Fibrezym 100 µg/ml, Suramin 0.2 µg/ml).

### Mouse bioassay

Ethics statement: When working with mice all efforts were made to minimize animal suffering. Inoculated animals were examined for neurological dysfunctions and euthanized (by carbon dioxide) when clinical signs became apparent. The animal experiments were approved and their conduction controlled by the Landesamt für Landwirtschaft, Lebensmittelsicherheit und Fischerei Mecklenburg-Vorpommern constituting the competent authority according to national and European legislation, namely the EU council directive 86/609/EEC on the protection of animals used for experimental and other scientific purposes (LVL M-V/310–4/7221.3–2.1–012/03). The competent authority follows the advice of an official animal welfare committee of the Federal State of Mecklenburg-Western Pomerania, Germany.

The in vivo infectivity assay was performed on a) wild-type C57BL/6 mice, b) Tgbov XV mice [43] and c) Tgshp XI mice [44]. In Tgbov XV mice the expression level of bovine PrP is 8 or 16 fold higher than that of the murine PrP in non-transgenic mice. In Tgshp XI mice the expression level of ovine PrP is 4 or 8 fold higher than that of the murine PrP in non-transgenic mice. The animals were infected intracerebrally with 30 µl of a 20% (wt/vol) cell solution (scrapie infected PES cells in PBS). Inoculated animals were examined for neurological dysfunctions and euthanized (by carbon dioxide) when clear clinical signs became apparent. TSE was confirmed detecting PrP^res^ by biochemical analysis (western-blot) in the brains of the diseased animals.

## RESULTS

### Selection of candidate cell lines for TSE infection studies

Cell lines were available to us from the collection of cell lines in veterinary medicine (CCLV) of the FLI, which deposits about 1400 different lines from a variety of eukaryotic species and tissues in various developmental stages. To obtain a cell culture model for native BSE or scrapie prions, we selected 54 of these cell lines. Primarily we chose cell lines of bovine, human, ovine and caprine origin. Additionally, we included cell lines that were derived from mink, cat, pig, deer, rabbit, hamster and mouse. The cells originated from the neural, endocrine, digestive, lymphatic, urinary or sexual system or were derived from musculature, bone marrow, connective tissue, epithelial cells or tumor cells. All 54 cell lines were analyzed repeatedly for their PrP^C^ expression levels. It became obvious that these were unique for each cell line and neither species specific nor tissue dependent. Finally 33 cell lines with easily detectable PrP^C^ levels were chosen for the infection experiments (Table 1).

### TSE infection studies on conventional neuronal and non-neuronal cell lines from different species

The 33 test cell lines were inoculated with bovine and mouse passaged BSE as well as ovine, caprine and mouse passaged scrapie brain homogenates and the clearance of the inoculum was monitored. Afterwards cells were examined for newly accumulated PrP^res^ by three different assays: Dot-blot analysis was used for primary screening, which allowed testing of a large number of samples with high sensitivity. Positive results were verified by western-blot analysis, which also provided information on the molecular weight and the glycosylation pattern of the detected PrP^res^. Finally the “Cell ELISA” was included to detect PrP^res^ at the single cell level. The majority of the inoculated cell lines did not propagate PrP^res^ following the challenge infection.

### Identification of a bovine cell line susceptible to natural sheep scrapie

**Figure 1:**
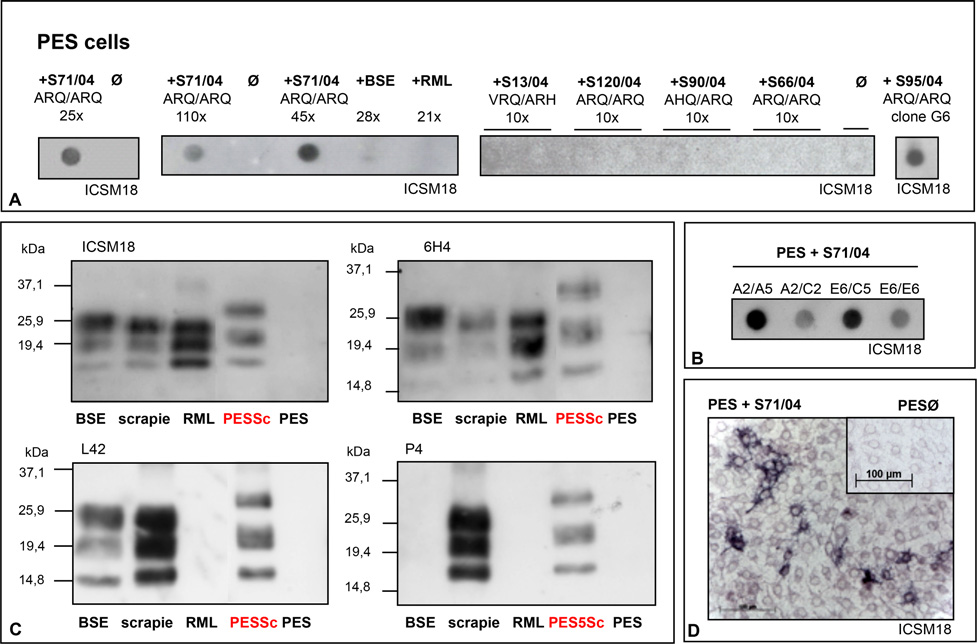
Persistent infection of a bovine cell line (PES) with sheep scrapie prions. **A)** PES cells were infected with the natural sheep scrapie isolates S71/04 and S95/04. Both isolates were derived from sheep carrying the ARQ/ARQ prion genotype. PrP^res^ was detected by dot-blot analysis. Shown here are samples from three independent infections with S71/04, assayed 25, 110 or 45 cell passages after inoculation. S95/04-infected PESSc cells were used for cell cloning and shown here is PESSc clone G6. Uninfected control cells (ᴓ) or PES cells challenged with BSE or RML prions that were passaged 28 and 21 times, respectively, show no detectable PrP^res^ signal. In addition the challenge with four other scrapie field isolates (S13/04, S120/04, S90/04 and S66/04) did not lead to PrP^res^ propagation; shown here are samples after 10 cell passages post inocula application. **B)** Serial cloning of PESSc cells that had been infected with scrapie isolate S71/04 resulted in cell populations with different PrP^res^ loads. Cell clones with stronger PrP^res^ signals were selected for further cultivation. **C)** Detecting PrP^res^ by western-blot PESSc prions were recognized by a panel of four different antibodies (ICSM18, 6H4, L42, P4) and show a higher molecular weight compared with BSE, scrapie and RML brain homogenate. **D)** The Cell-ELISA confirmed the infection of PES cells, showing PrP^res^ positive single cells and cell accumulations. Uninfected PES cells are shown in the inlet.

Among the 33 test cell lines there were also three bovine MDBK cell lines that were challenged with BSE and scrapie prions. While two remained uninfected, MDBK sub-line PES was found to be susceptible to natural sheep scrapie but not to BSE prions. The prnp gene of PES cells was verified as bovine six-octarepeat sequence encoding for 264 amino acids. The inoculum that was used for the infection was a field-isolate (S71/04 ARQ/ARQ) that originated from a German outbreak of classical sheep scrapie in 2004. After residual inoculum was completely removed, positive signals of PrP^res^ were detected by dot-blot analysis, western-blot analysis and the “Cell ELISA” using a variety of antibodies (Fig. 1). Up to the 10^th^ passage only low levels of PrP^res^ had been detected, but the signal increased over time and with further passages. After more than 200 passages the cells still continued to propagate PrP^res^. The infection was repeated several times. Selective cloning efforts increased the infection level (determined by PrP^res^-load) of the infected cell cultures (Fig. 1A and 1B). The susceptibility of PES cells to prions was limited to certain scrapie isolates (Fig. 1A). While challenged with brain homogenates from various classical and atypical sheep scrapie cases as well as with homogenates from classical and atypical cattle-derived BSE samples (see materials and methods), only five different sheep scrapie isolates (S11/04, S67/04, S69/04, S71/04, S95/04 ARQ/ARQ), all but one from the same affected flock, were found to be infectious for PES cells.

Proteinase K (PK) treatment and electrophoretic separation showed that PrP^res^ derived from scrapie infected PES (PESSc) cells—especially the two bands that represent the mono- and the diglycosylated form of PrP—had a higher molecular weight than PrP^res^ derived from brain homogenates of BSE diseased cattle, from mice that were experimentally infected with RML scrapie or from the sheep scrapie isolate that had been used to infect the PES cells (Fig. 1C). Yet, identically to sheep scrapie prions, PESSc prions were recognized by a panel of four different antibodies: ICSM18, 6H4, L42 and P4 (Fig. 1C), as opposed to cattle-derived BSE prions that are not recognized by P4 [45], or to murine RML prions that are neither detected with P4 nor L42 [37, 46].

**Figure 2:**
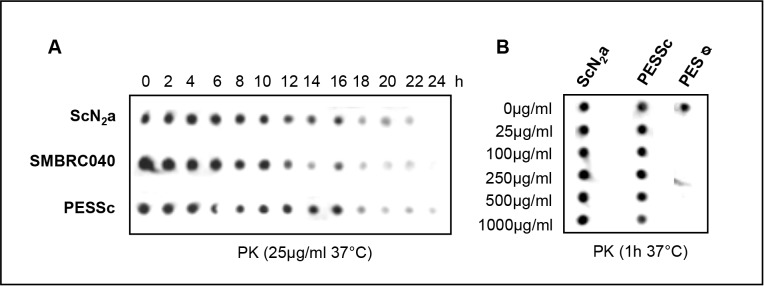
Relative resistance of PESSc prions to ProteinaseK. **A)** Cell lysates from PESSc cells, ScN2a cells and SMBRC040 cells were each divided into 13 aliquots. All aliquots, but one of each cell line, were subjected to ProteinaseK digestion (25 µg/ml; at 37°C). Every two hours the digestion of one aliquot per cell line was stopped with PMSF. PrP^res^ was detected by dot-blot analysis (mAB ICSM18). In both murine cell lysates PrP^res^ was detectable for up to 22 hours; in PESSc cell lysates PrP^res^ was still detectable after 24 hours. **B)** Cell lysates from uninfected PES cells, PESSc cells and ScN2a cells were divided into six aliquots each, ProteinaseK was added at increasing concentrations (0 µg/ml, 25 µg/ml, 100 µg/ml, 250 µg/ml, 500 µg/ml and 1000 µg/ml), and the samples were incubated for one hour at 37°C. The digestion was terminated by adding PMSF. PrP^res^ was detected by dot-blot analysis (mAB ICSM18). While 25 µg ProteinaseK/ml were sufficient to clear the control lysate from any detectable PrP, strong PrP^res^ signals were detected in both prion infected samples up to 500 & 1000 µg ProteinaseK/ml.

When treated with either increasing concentrations of PK or with the same concentration of PK over an increasing period of time, PESSc prions were found at least equally resistant to protease digestion as prions of RML infected ScN_2_a and SMBRC040 cells (Fig. 2).

**Figure 3:**
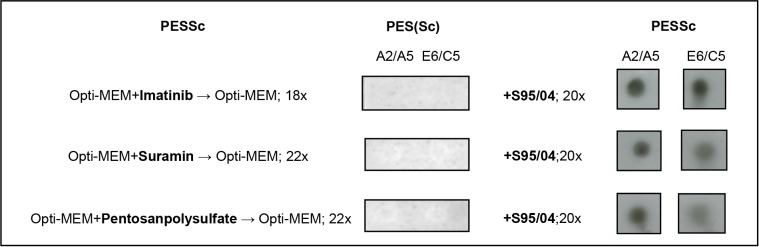
Re-infection of „cured“ PESSc cells with different sheep scrapie field isolate. PESSc cell clones A2/A5 and E6/C5, propagating ovine scrapie prions from the sheep scrapie field isolate S71/04, had been cured with the prion inhibitors Imatinib, Suramin and Pentosanpolysulfat until any detectable PrP^res^ was cleared from the culture. Following that, the cells were cultured for 18 to 22 splits in the absence of the inhibitors and remained uninfected as monitored by dot-blot analysis. The cells were then subjected to infection with a second ovine scrapie field isolate, S95/04, and split 20 times at a ratio of 1:2. PrP^res^ was detected by dot-blot analysis. mAB: ICSM18.

Imatinib (10µM), Pentosanpolysulfat (100 µg/ml) and Suramin (0.2 µg/ml), were previously reported to cure prion infected ScN_2_a and SMBRC040 cells [40–42]. In our experiments, PrP^res^ signal in ScN_2_a and SMBRC040 cells was strongly decreased after a four-day treatment with these compounds and cells were cured after eleven days. In PESSc cells clearance of PrP^res^ took four weeks with Pentosanpolysulfat or Suramin and up to four month with Imatinib. Cured cells were still susceptible. They could be re-infected with either the initial or a different sheep scrapie isolate (Fig. 3).

**Figure 4:**
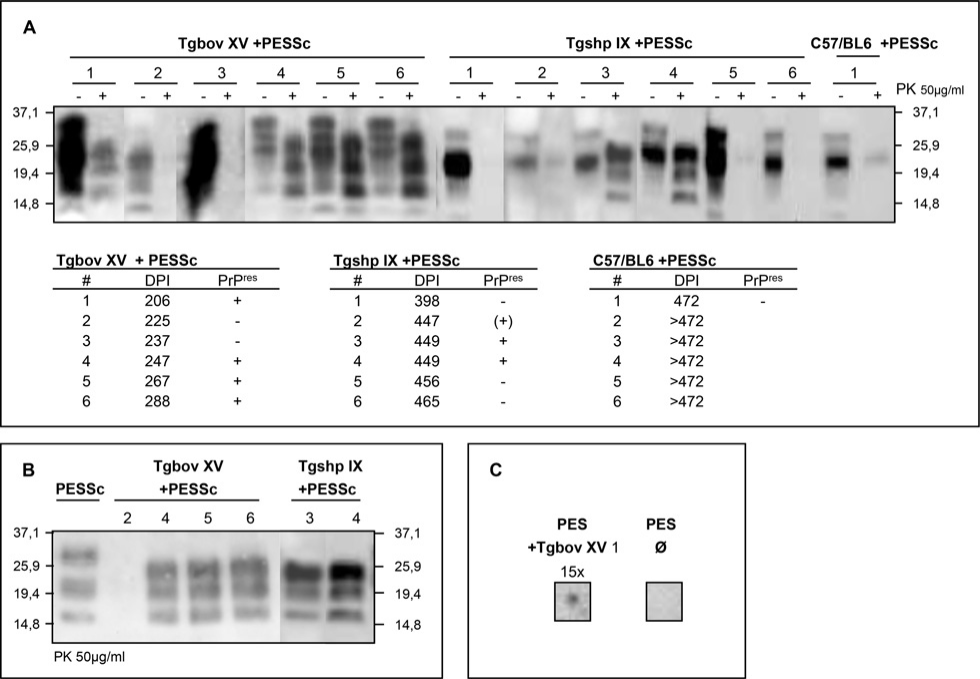
Verification of infectivity in PES cells by mouse bioassay. **A)** Cell lysates of PESSc cells were inoculated into three groups of mice (six animals each group): Tgbov XV, Tgshp IX and C57/BL6. Tgbov XV mice succumbed to disease 245 ± 29 dpi, Tgshp IX mice died 444 ± 23 dpi and the first non-transgenic mouse was culled due to clinical signs 472 days post inoculation. The brain homogenates were tested by western-blot analysis for PrP^res^ signals. **B)** Side by side western-blot analysis of PK digested PrP^res^ derived from PESSc cells shows a higher molecular weight than PK digested PrP^res^ from PESSc infected Tgbov XV or Tgshp IX mice. **C)** PES cells were inoculated with brain homogenate from Tgbov XV mouse 1. The cells were split 15 times at a ratio of 1:2 and were then assayed for PrP^res^ by dot-blot analysis. Uninfected PES cells were assayed as control Ø. mAB: ICSM18.

To verify a productive infection in PrP^res^ producing PES cells, cell homogenates were bioassayed by intracerebral inoculation in a) transgenic mice overexpressing bovine PrP^C^ (Tgbov XV), b) transgenic mice overexpressing ovine PrP^C^ (Tgshp IX) and c) wild-type C57BL/6 mice. All inoculated Tgbov XV mice succumbed to scrapie with typical clinical signs after incubation times of 245 ± 29 days. Interestingly, PrP^res^ detected by western-blot analysis in the brains of these mice (Fig. 4A and 4B) resembled the PrP^res^ in the original brain-derived inoculum, i.e. had a lower molecular weight after PK cleavage (Fig. 4B). Moreover these Tgbov XV mouse brains were successfully used to transiently re-infect PES cells (Fig. 4C). The Tgshp IX mice succumbed to scrapie with incubation times of 444± 23 days and two out of six mice were tested positive for PrP^res^ by western-blot analysis (Fig. 4A and 4B). The incubation time in the C57BL/6 mice exceeded 470 days.

## DISCUSSION

We have tested a collection of conventional eukaryotic cell lines for their susceptibility to ruminant-derived BSE and scrapie prions. In accordance to earlier studies with experimental rodent adapted prion strains most cell lines resisted our efforts and could not be infected.

Surprisingly, the most promising cell line that we found was a sub-line of the well-established and commonly used bovine kidney cell line MDBK [47], designated PES. As described for other cell sub-lines previously [7, 22], we found that MDBK cell lines vary in their susceptibility towards prion infection as well. PES cells were reliably and repeatedly infected with natural sheep scrapie prions, while two other MDBK test cell lines remained uninfected.

As expected, early after inoculation, with only a few PES cells being infected, signals were too low to be detected. However, with further passages and the infection spreading throughout the cell population, signals became strong. They remained steady even after more than 200 passages, indicating that there was no essential up or down regulation of cellular factors critical for prion amplification. As described for other prion cell culture models, only a small part of the PES cell population became infected or at least propagated PrP^res^ at detectable levels. This heterogeneity among the cells might indicate that certain cells are susceptible to infection, while others are resistant, or they might just differ in efficacy of replication. Small variations in the rate of protein synthesis or protein degradation could either permit productive prion replication or cause the clearance of the infection in the culture [48]. Frequently discussed are possible differences in availability or abundance of fitting PrP^C^ conformations within a cell specific PrP^C^ repertoire or other necessary but still unknown cofactors that might influence host-agent correspondence or cell metabolism [49, 50]. In addition, serial cloning resulted in higher infected sub-lines. However, even in cloned cell lines only 20–30% of the culture retained a detectable PrP^res^ level. Similar observations were made in previously reported studies [22, 29, 34]. Ongoing clearance from scrapie infection must be balanced in some way by prion replication, division of infected cells and possibly the new infection of yet uninfected but susceptible cell clones.

The actual infectivity of PESSc cells was verified by mouse bioassay using transgenic Tgbov XV and Tgshp XI mice overexpressing bovine or ovine PrP^C^ respectively as well as by inoculating conventional C57BL/6 mice. As expected, due to the bovine nature of the host cell line, shortest incubation times were observed in bovinized transgenic mice followed by ovinized and wild-type mice.

There is no cell culture model for natural BSE prions available to date. Therefore it was not surprising that PES cells, though being of bovine nature, were only infected by scrapie but not by BSE prions. Since sheep-derived scrapie prions have to cross the species barrier to cattle, higher infection doses might be needed for a successful infection. This explains perhaps that not all inocula coming from sheep scrapie cases belonging to a single given flock, even when the sheep carried the same genotype, were used successfully for the infection of PES cells. Another, far more likely, possibility would be that this particular flock was actually infected with more than one scrapie strain. PES cells might have presented a particular good environment for one strain, while being disadvantageous for another.

We hypothesize that PES cells not only offered a particular good environment for a specific scrapie strain, but may also have selectively propagated a previously rather minor constituent of that strain’s prion population [23, 49–52]. According to a panel of four different antibodies PESSc prions were indistinguishable from sheep scrapie prions, but distinct from RML or cattle-derived BSE [45, 46, 53, 54]. The molecular weight of PESSc prions after PK digestion, however, was peculiarly large when compared with brain-derived prions, including those derived from the original sheep scrapie inoculum. Especially striking were the two upper bands, representing the mono- and the diglycosylated form of the prion protein, but even the unglycosylated band was slightly elevated compared with the brain-derived samples. While the distinct physicochemical properties of prion strains, i.e. a strain’s relative resistance to ProteinaseK digestion [45, 53, 54], are thought to be enciphered in the conformation of its PrP^Sc^ [55], prions are also understood as quasi-species [56] comprised of constantly arising mutants from which the fittest in a particular environment is propagated as the major component of the population [50, 51]. Fitness in the present case could have been conformational similarity to the host’s own PrP^C^ repertoire [49, 50]; if and to what extend posttranslational modifications such as glycosylation or the association with cellular components contribute to this selection process is still debated [57].

After PESSc prions had been propagated in mice, we found that the molecular weight of PK digested PrP^res^ derived from these mice was again distinct from that of the cell-derived inoculum. It seemed reverted into a brain adapted version [51, 52, 58, 59] and may or may not represent the same population as the original sheep scrapie strain.

We do not know whether the five successfully inoculated scrapie field isolates contained the same prion strain or each one contained a different strain while sharing the same sub-strain. It is also possible that PES cells are able to propagate more than one particular sub-strain. Notably, PES cells are permissive for scrapie isolates from sheep that carried the prion genotype ARQ/ARQ. So far, all available cell lines that are susceptible to sheep scrapie field isolates are permissive for VRQ/VRQ and ARR/VRQ genotypes, but failed to propagate isolates of ARQ/ARQ genotypes [60]. We believe that PES cells used together with other prion permissive cell lines [7, 60, 61] will prove a valuable tool for ongoing efforts to understand prions and prion diseases.
