# Correction: A Bovine Cell Line That Can Be Infected by Natural Sheep Scrapie Prions

**DOI:** 10.1371/journal.pone.0121881

**Published:** 2015-03-25

**Authors:** The PLOS ONE Staff

The sixth author’s name is spelled incorrectly. The correct name is: Herman Schatzl. The correct citation is: Oelschlegel AM, Geissen M, Lenk M, Riebe R, Angermann M, Schatzl H, et al. (2015) A Bovine Cell Line That Can Be Infected by Natural Sheep Scrapie Prions. PLoS ONE 10(1): e0117154. doi: 10.1371/journal.pone.0117154
